# Early therapy evaluation of intra-arterial trastuzumab injection in a human breast cancer xenograft model using multiparametric MR imaging

**DOI:** 10.1371/journal.pone.0300171

**Published:** 2024-05-03

**Authors:** Bo Kyu Kim, Byungjun Kim, Sung-Hye You, Moon-Sun Jang, Geun Ho Im, Keon-Ha Kim

**Affiliations:** 1 Department of Radiology, Korea University Anam Hospital, Seoul, Korea; 2 Department of Radiology, Samsung Medical Center, Sungkyunkwan University School of Medicine and Center for Molecular and Cellular Imaging, Samsung Biomedical Research Institute, Seoul, Korea; 3 Center for Neuroscience Imaging Research, SungKyunkwan University, Suwon, Korea; 4 Department of Radiology, Samsung Medical Center, Sungkyunkwan University School of Medicine, Seoul, Korea; Memorial Sloan Kettering Cancer Center, UNITED STATES

## Abstract

**Purpose:**

To investigate the treatment efficacy of intra-arterial (IA) trastuzumab treatment using multiparametric magnetic resonance imaging (MRI) in a human breast cancer xenograft model.

**Materials and methods:**

Human breast cancer cells (BT474) were stereotaxically injected into the brains of nude mice to obtain a xenograft model. The mice were divided into four groups and subjected to different treatments (IA treatment [IA-T], intravenous treatment [IV-T], IA saline injection [IA-S], and the sham control group). MRI was performed before and at 7 and 14 d after treatment to assess the efficacy of the treatment. The tumor volume, apparent diffusion coefficient (ADC), and dynamic contrast-enhanced (DCE) MRI parameters (Ktrans, Kep, Ve, and Vp) were measured.

**Results:**

Tumor volumes in the IA-T group at 14 d after treatment were significantly lower than those in the IV-T group (13.1 mm^3^ [interquartile range 8.48–16.05] vs. 25.69 mm^3^ [IQR 20.39–30.29], p = 0.005), control group (IA-S, 33.83 mm^3^ [IQR 32.00–36.30], p<0.01), and sham control (39.71 mm^3^ [IQR 26.60–48.26], p <0.001). The ADC value in the IA-T group was higher than that in the control groups (IA-T, 7.62 [IQR 7.23–8.20] vs. IA-S, 6.77 [IQR 6.48–6.87], p = 0.044 and vs. sham control, 6.89 [IQR 4.93–7.48], p = 0.004). Ktrans was significantly decreased following the treatment compared to that in the control groups (p = 0.002 and p<0.001 for vs. IA-S and sham control, respectively). Tumor growth was decreased in the IV-T group compared to that in the sham control group (25.69 mm^3^ [IQR 20.39–30.29] vs. 39.71 mm^3^ [IQR 26.60–48.26], p = 0.27); there was no significant change in the MRI parameters.

**Conclusion:**

IA treatment with trastuzumab potentially affects the early response to treatment, including decreased tumor growth and decrease of Ktrans, in a preclinical brain tumor model.

## Introduction

The incidence of brain metastasis in patients with metastatic breast cancer has increased over time, and the importance of early detection and treatment of lesions in prolonging overall survival has been emphasized [1]. Various systemic therapeutic options exist for metastatic breast cancer, depending on the hormone receptor and human epidermal growth factor receptor (HER2) status. However, despite these treatments, the median overall survival is still low in patients with brain metastases; it is approximately 3–30 months [2, 3]. Systemic chemotherapy has limited efficacy in brain metastasis because of several factors: difficulty in the chemotherapeutic agents crossing the blood-brain barrier (BBB), the expression of drug outflow pumps in the BBB, and the acquired resistance of metastatic cancer against anticancer drugs [4].

Trastuzumab (Herceptin, Genentech, San Francisco, CA, USA) is a monoclonal antibody against the HER2, and it is used as a standard treatment since it was approved for HER2-overexpressing metastatic breast cancer in 1998 [5, 6]. According to RegistHER, a prospective observational study of 1023 patients with HER2-positive metastatic breast cancer, the median overall survival for patients treated with trastuzumab was 17.5 months from the day of diagnosis of central nervous systsem metastasis; patients who did not undergo trastuzumab treatment had a median overall survival of 3.8 [7]. Similar to other macromolecules, trastuzumab has difficulties passing through the BBB. BBB is an important impediment to the delivery of chemotherapeutic agents in animal models of brain metastasis from breast cancer [8]. In the same experiment, the drug concentration in peripheral metastatic lesions was at least 10-fold higher than that in brain metastasis [8]. For this reason, the incidence of brain metastasis of HER2-positive breast cancer increases along the prolonged survival with well-controlled extracranial metastatic lesions [9, 10]. Intra-arterial (IA) treatment is one of the strategies can improve the delivery of therapeutic agents to brain metastasis by increasing the local plasma peak concentration compared to intravenous (IV) treatment; in addition, it reduces systemic side effects [10, 11]. Animal studies have shown that IA infusion can increase the local concentration by approximately 3 to 5.5 times compared to IV infusion [12, 13].

Magnetic resonance imaging (MRI) is important for evaluating the response to treatment in brain tumors. MRI provides information on peritumoral edema, cellular density, vascular perfusion, permeability, and tumor morphology. Gliomas show an increase in the apparent diffusion coefficient (ADC) before tumor volume changes following cytotoxic therapy; this is attributed to a decrease in tumor cellularity [14, 15]. In addition, dynamic contrast enhancement (DCE) perfusion studies can be used to evaluate the vascular response of tumors, which show changes with anti-angiogenic or radiation therapy before tumor volume changes [16–20]. Therefore, this study used multiparametric MRI to evaluate the efficacy of IA treatment in a mouse brain metastasis xenograft model of human breast cancer and the early tumor response (within 3 weeks after treatment).

## Materials and methods

### In vitro study

To identify a suitable cell line for the experiment, we evaluated the treatment efficacy using a human breast cancer cell line expressing HER2, which was trastuzumab-responsive in vitro. Cell lines (BT474, ZR75-1, and SKBR3) was obtained from the American Type Culture Collection and were cultured in Hybri-Care medium, RPMI-1640 medium, and McCoy’s 5a medium, respectively. Each cell line treated with 10% fetal bovine serum mixed with 100 U/mL penicillin and 100 U/mL streptomycin, and was incubated in a humidified incubator containing 5% CO_2_ at 37°C. For the in vitro viability assay of trastuzumab, cells were cultured in 96-well plates (1x10^4^ cells/well) for 24 h and incubated with different concentration of trastuzumab diluted with saline (1, 3, 5, 10, 25, 50, 100, 250, 500, 1000 μg/mL). After incubation, 10 μL of CCK-8 solution (Dojindo Laboratories, Kumamoto, Japan) was added, and cultured 1 h. The absorbance of each well at 450 nm was measure with a microplate spectrophotometer (xMrak, BIO_RAD). The cell counts of each cell line before and after trastuzumab treatment were compared.

### Animals

This animal experiments were reviewed and approved by the Institutional Animal Care and Use Committee (IACUC) of Samsung Medical Center. (Approval No. 20180706001) We did not obtain ethical approval because our study does not include human participants or human subjects’ data. Seven-week-old female BALB/c-nu mice (KCLB, Seoul, South Korea) with a body weight of 20.0 ± 1.2 g were used for developing the tumor xenograft models. Total 40 mice were used in this study. Mice were housed in a barrier facility with high-efficiency particulate air filtration and fed an autoclaved laboratory diet. During all the surgical procedures and MRI acquisition, the mice were anesthetized through inhalation of isoflurane (5% for induction and 1–1.5% during the procedure and MR experiment) in a mixture of O_2_ and N_2_ (3:7) via a nose cone, allowing spontaneous respiration. The animals were weighed and monitored daily to evaluate any adverse event. Animals with significant clinical observations of morbidity (eg. body weight loss > 30%, inactivity, dyspnea) were euthanized within 24 hours. Humane endpoint used in this study is described in S1 File. The treatments for the other mice were terminated three weeks after the date randomization, and they were euthanized.

### Tumor xenograft model

**Fig 1:**
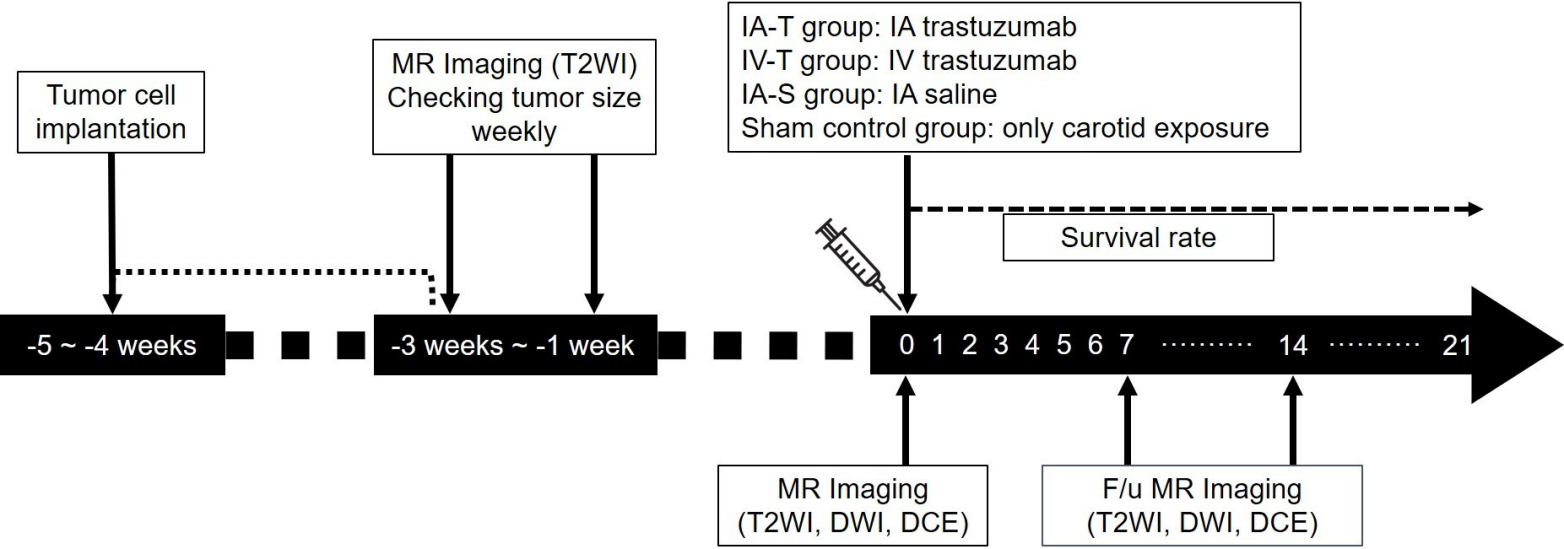
Flow diagram of the study. After tumor implantation, tumor size was monitored using T2WI MRI weekly. After the largest tumor dimension reached 1.5 ± 0.5 mm on coronal image of T2WI, baseline MRI was performed after 7 days. Treatment was performed according to the randomized group at the day of baseline MRI. (Day 0).

Each cell line was inoculated into the brain of mice using a stereotaxic instrument (Benchmark digital stereotax; Leica Biosystems). A small hole was drilled in the skull using a 31-gauge needle. A total of 3 μL of tumor cells (1 × 10^5^) were injected into the right caudate nucleus at 0.14 mm anterior to the bregma, 2.0 mm right lateral to the bregma, and 3.0 mm depth from the dura. Tumor volume was monitored using T2WI MRI weekly starting 2 weeks after tumor inoculation. After the largest tumor dimension reached 1.5 ± 0.5 mm on T2WI, baseline MRI including T2WI, DWI, and DCE performed after 7 days. And treatment was performed according to the randomized group after baseline MRI. The day on which treatment was performed was set as day 0. The flow chart of MRI schedule is demonstrated in Fig 1.

### Drug preparation

Trastuzumab (Herceptin; Roche, Basel, Switzerland) is a humanized IgG1 monoclonal antibody directed against the extracellular domain of HER2. Trastuzumab was stored at 4°C and reconstituted with sterile saline; the solution was diluted to 8 mg/kg for a total volume of 10 μL.

### Animal treatments

The animals were randomized into four groups based on body weight 3–4 weeks after tumor implantation. The IA-T group received trastuzumab via the IA route with 8 mg/kg trastuzumab. In contrast, the IV-T group received 8 mg/kg trastuzumab intravenously. To assess the risk of the IA injection, an equal volume of saline was injected into the IA-S group via the IA route. The sham control group underwent skin incision and carotid artery exposure without IA or IV injection.

The IA injection was performed under anesthesia described above. A 10 mm longitudinal midline incision was made from the hyoid bone in the sternal notch direction. The carotid sheath was exposed by lateral retraction of the sternocleidomastoid muscle and medial retraction of the sternohyoid muscle. After careful blunt dissection, the right common carotid artery (CCA), carotid bifurcation, and proximal internal and external carotid arteries were exposed. The pterygopalatine artery, which arises from the internal carotid artery as a lateral branch, was ligated to accurately navigate the intracranial injection. The distal CCA was cannulated using a polyethylene tube (Becton Dickinson) with an outer diameter of 0.40 mm. With precise manipulation, the heart-shaped tip of the tube was navigated to the distal internal carotid artery, passing through the ligated pterygopalatine artery, and a single injection of trastuzumab solution or saline was administered. The CCA was ligated proximal to the cannulation site after removing the cannula. The intravenous injection was performed by puncturing the tail vein using a 31-gauge butterfly needle, followed by the injection of a trastuzumab.

### MR image acquisition

MRI was performed on day 0 to confirm the adequate tumor volume and a follow up MRI was performed on day 7 and day 14 after the treatment.

MRI was performed using a 7T USR Preclinical MR system (Bruker-Biospin, Fallanden, Switzerland). A quadrature birdcage coil (Bruker Biospin; 72 mm inside diameter) and an actively decoupled phased-array coil were used for excitation and signal reception, respectively. Coronal T2-weighted images (T2WIs) were obtained with a high-spatial resolution fast spin echo sequence (RARE); TR = 2500 ms, TE = 50 ms, NEX = 6, echo train length = 8, flip angle = 90°, slice thickness of 700 μm without interslice gap, matrix size = 160 × 160, and a field of view = 16 × 16 mm. The diffusion-weighted image was acquired with a spin-echo planar imaging (EPI) sequence: TR = 3000 ms, TE = 34 ms, NEX = 4, echo train length = 21, flip angle = 90° slice thickness of 700 μm, matrix size = 256 × 126, a field of view = 16 × 16 mm, and a b value of 0, 200, 400, 800, and 1200 s/mm^2^. Twelve and four coronal slices of T2WIs were acquired separately for exact volume measurement and co-registration with DCE-MRI images, respectively.

For DCE-MRI imaging, four coronal slices were acquired using the T1-weighted fast low-angle shot sequence (FLASH) in the same plane as T2WI. The specific imaging parameters as follow; TR = 20 ms, TE = 2 ms, NEX = 1, flip angle = 25°, number of repetitions = 300, slice thickness of 700 μm without interslice gap, matrix size = 160 × 80, and field of view = 16 × 16 mm. The temporal resolution was 0.6 sec, and the total acquisition time of DCE-MRI was 8 min. After pre-contrast T1WI acquisition, a catheter placed within the tail vein delivered 0.1 mmol/kg of gadoterate meglumine (Dotarem, Guerbet, France) over 2 s via the injector. For T1 mapping, four coronal slices of pre-contrast T1WI were obtained using the same parameters with flip angles of 5°, 15°, 35°, 45°, 60°, and 70°.

### Histopathology

For immunohistochemical staining of CD31 and Ki-67, The isolated brains were fixed overnight in 4% formalin solution at room temperature, and embedded in paraffin. Next, the embedded brain tissues were serially sectioned in the coronal plane at a thickness of 5 μm. The sections were incubated with anti-CD31 (cell signaling technology) and Ki-67(novus biologicals) rabbit polyclonal antibody diluted 1:100 overnight at 4°C. After washing in PBS, the sections were incubated for 1 h at room temperature with HRP-labeled polymer-conjugated secondary antibody against rabbit IgG (DAKO). Finally, the sections were detected using the ready-to-use DAB (3,3’-diaminobenzidine) substrate chromogen solution (DAKO) for 10 minutes. Finally, the sections were stained with hematoxylin and eosin.

The image of high-power field (HPF) (objective 40 X) of each selected hot-spot was taken from representative cases. ImageJ platform (http://rsb.info.nih.gov/ij/index.html) was used to precisely count brown and blue stained nuclei. The image was postprocessed with image binarization and cell number counted manually.

### Image analysis

Image analysis was performed using a commercially available software package (Nordic ICE, NordicNeuroLab, Bergen, Norway). The tumor volume was measured by manually drawing the region of interest (ROI) using the 12-slice T2WI. To apply the ROI to the other sequences, each image sequence was automatically co-registered with T2WI (S2 File).

The perfusion analysis first normalized the DCE image using T1 mapping with six series of T1WI with different flip angles. We used a population-based arterial input function (AIF) derived separate cohort of tumor bearing mice in our preliminary study for incorporation the variation of indivisuals and yielding high singal-to-noise ratio. The measurement of population-based AIF described in the S3 File. The Extended Toft model (Eqs [1] and [2]) was used to calculate Ktrans, Kep, and Ve for each voxel [21].

$$C_{t}(t)=v_{p}C_{p}(t)+K^{trans}\int_{0}^{t}C_{p}(\tau )exp[-\frac{K^{trans}(t-\tau )}{v_{e}}]d\tau$$ [1]

Kep=Ktrans/Ve [2]

where Ct and Cp are the contrast agents in the tissue and plasma space, respectively; Ktrans is the volume transfer constant and Ve is the extravascular extracellular volume fraction. Voxels for which the fitting routine yielded non-physiological values (Ktrans < 0.01 min^-1^, Ktrans > 5 min^-1^, Ve > 1) were eliminated from further analyses.

### Statistical analysis

Statistical analyses were performed using SPSS software version 22.0 (SPSS, Chicago, IL, USA). The data were analyzed using repeated-measures analysis of variance (ANOVA) to compare ADC values ​​and DCE parameters by time in each group. Post hoc analyses using Bonferroni correction were performed to assess the differences in the pharmacokinetic parameters among the four groups. When a normal distribution was not satisfied, the Kruskal-Wallis test was performed. If significant results were obtained from the Kruskal-Wallis test, the Mann-Whitney test was performed as a post hoc analysis. All data are represented as median with interquartile range (IQR); a P value less than 0.05 was considered significant.

## Results

### In vitro study

In the in vitro study, more than 30% cell death was observed for BT474 from 1 μg/mL concentration of trastuzumab, and no cell death was observed for ZR75-1 that treated <10 μg/mL. In the case of SKBR3, approximately 20% cell death was observed at <10 μg/mL; therefore, BT474 was considered the most sensitive cell line (S1 Fig).

In a preliminary study assessing the inoculation of each cell line, the tumor growth with BT474 was more suitable for evaluation than that with SKBR3 or ZR75-1. SKBR3 and ZR75-1 showed tumor sizes of less than 1 mm on T2-weighted images taken after 4 and 6 weeks of inoculation, respectively. In contrast, the maximum diameter of BT474 was 1.60 ± 0.38 at 4 weeks and 2.40 ± 0.51 at 6 weeks. During the 6-week tumor growth period, a few individuals showed increased internal tumor necrosis; therefore, treatment was performed during the 4^th^ or 5^th^ week of inoculation (S2 Fig).

### Treatment groups and survival rates

**Fig 2:**
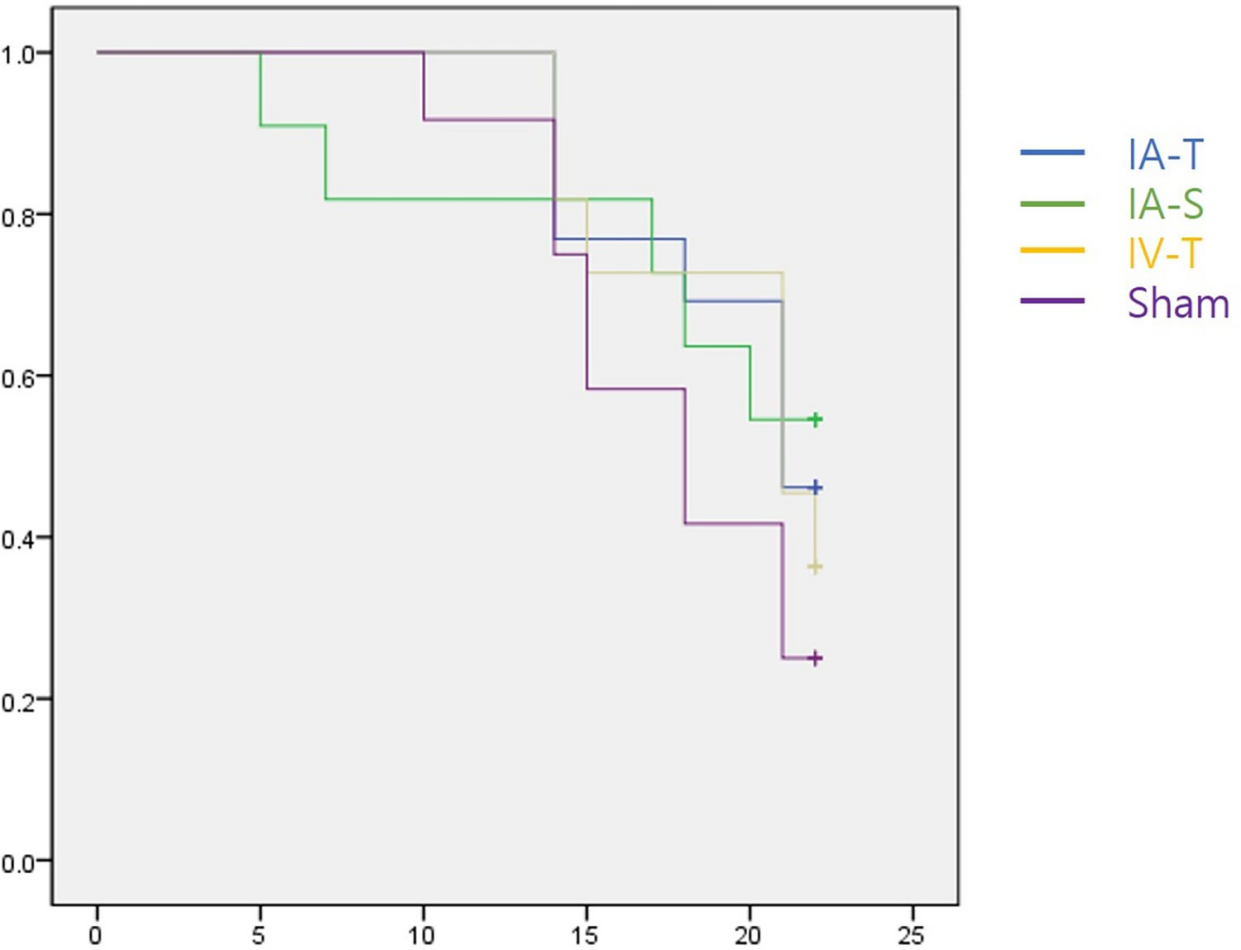
Kaplan-Meier survival plots of treatment groups. Each treatment group is demonstrated as follows: Intra-arterial trastuzumab treatment (IA-T, n = 10, blue line), intravenous chemotherapy treatment (IV-T, n = 10, yellow line), intra-arterial saline injection (IA-S, n = 10, green line), and sham control groups (Sham, n = 10, purple line). Survival rates were 17.5 ± 6.3 days (IA-T), 16.9 ± 7.9 days (IA-S), 19.6 ± 3.4 days (IV-T), and 17.7 ± 4.0 days (Sham control). No significant differences were observed among the four groups. (p = 0.491).

The treatment groups were randomly assigned according to body weight measured 3–4 weeks after tumor inoculation. All animals survived after stereotaxic tumo rinoculation until treatment began. Total 35 mice survived until the end of the experiment. Five mice (IA-S group: 2, IV-T group: 1, Sham control group: 2) euthanized before day 14 because of excessive tumor growth and were excluded from further image analysis. Survival rates were 17.5 ± 6.3 days for the IA-T group, 16.9 ± 7.9 days for the IA-saline group, 19.6 ± 3.4 days for the IV-T group, and 17.7 ± 4.0 days for the Sham control group. The difference between the groups, analyzed using Kaplan-Meier survival analysis, was insignificant (p = 0.491) (Fig 2).

### Tumor volume and MRI parameter analysis

**Fig 3:**
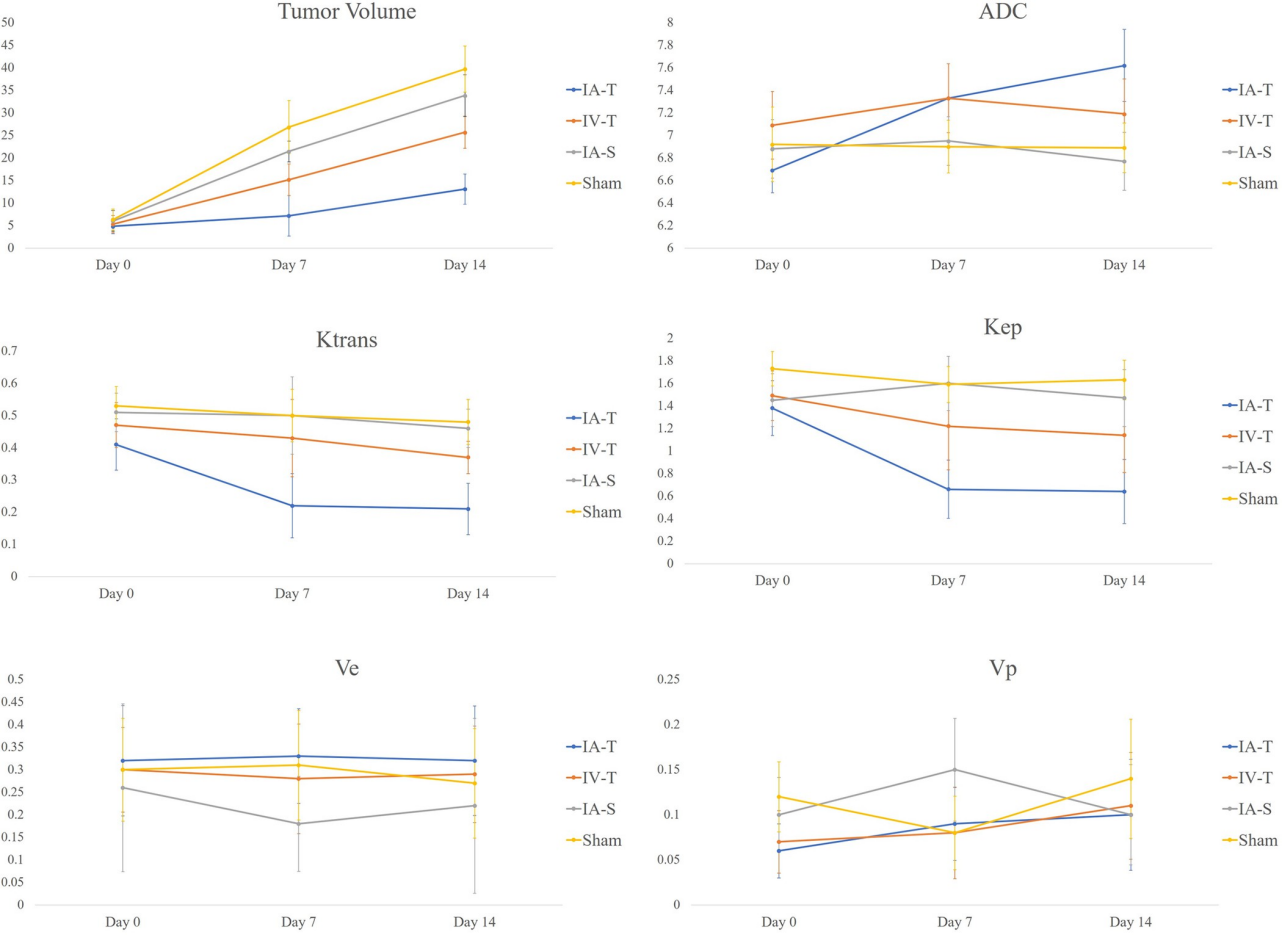
Changes in tumor volume and DCE parameters. Changes in tumor volume, ADC value, and DCE parameters for four groups (IA-T, n = 10; IV-T, n = 9; IA-S, n = 8; Sham, n = 8) on days 7 and 14. The IA-T and IV-T groups showed a decrease in tumor growth compared to the control group, and only the IA-T group showed a statistically significant reduction in tumor growth on day 14. Significant increases in ADC values and decreases in Ktrans and Kep, compared to that in the control groups, were observed only in the IA-T group.

The tumor volume and MRI parameters of each group, measured on days 0, 7, and 14, are summarized in Table 1 and Fig 3. On day 7, tumor growth was significantly reduced in both groups receiving trastuzumab treatment compared to that in the control group (For IA-T, 7.18 mm^3^ [IQR 7.08–12.50] vs. IA-S, 21.44 mm^3^ [IQR 19.23–25.50], p<0.001; vs. Sham control, 26.83 mm^3^ [IQR 23.52–29.01], p <0.001; For IV-T, 15.20 mm^3^ [IQR 9.81–19.04], p = 0.013 when compared to IA-S and p <0.001 when compared to Sham control). On day 14, the IA-T group showed even lower tumor growth when compared to that in the IV-T group (13.1 mm^3^ [IQR 8.48–16.05] vs. 25.69 mm^3^ [IQR 20.39–30.29], p = 0.005).

**Table 1:** Summary of tumor volumes and imaging parameters.

		Group				p-value^a^					
		IA-T (n = 10)	IV-T (n = 9)	IA-S (n = 8)	Sham control (n = 8)	IA-T vs IV-T	IA-T vs IA-S	IA-T vs Sham	IV-T vs IA-S	IV-T vs Sham	IA-S vs Sham
Tumor volume (mm^3^)	Day 0	4.85 (3.56–7.70)	5.33 (3.86–6.71)	6.04 (3.52–9.65)	6.35 (5.56–7.91)	1.000	1.000	1.000	1.000	1.000	1.000
	Day 7	7.18 (7.08–12.50)	15.20 (9.81–19.04)	21.44 (19.23–25.50)	26.83 (23.52–29.01)	0.188	<0.001	<0.001	0.013	<0.001	0.287
	Day 14	13.1 (8.48–16.05)	25.69 (20.39–30.29)	33.83 (32.00–36.30)	39.71 (26.60–48.26)	0.005	<0.01	<0.001	0.314	0.027	1.000
ADC (10^-4^mm^2^/s)	Day 0	6.69 (6.69–7.23)	7.09 (6.66–7.65)	6.88 (6.71–7.57)	6.92 (5.77–7.89)	1.000	1.000	1.000	1.000	1.000	1.000
	Day 7	7.33 (6.85–7.83)	7.33 (6.76–7.93)	6.95 (6.39–7.13)	6.90 (5.16–7.99)	1.000	1.000	0.523	1.000	0.444	1.000
	Day 14	7.62 (7.23–8.20)	7.19 (6.88–7.48)	6.77 (6.48–6.87)	6.89 (4.93–7.48)	0.806	0.044	0.004	1.000	0.245	1.000
Ktrans (min^-1^)	Day 0	0.41 (0.29–0.69)	0.47 (0.30–0.66)	0.51 (0.30–0.65)	0.53 (0.40–0.58)	1.000	1.000	1.000	1.000	1.000	1.000
	Day 7	0.22 (0.11–0.37)	0.43 (0.20–0.56)	0.50 (0.27–0.60)	0.50 (0.44–0.54)	0.704	0.126	0.015	1.000	0.716	1.000
	Day 14	0.21 (0.07–0.26)	0.37 (0.30–0.43)	0.46 (0.34–0.55)	0.48 (0.38–0.080)	0.049	0.002	<0.001	1.000	0.132	0.921
Ve	Day 0	0.32 (0.22–0.37)	0.30 (0.25–0.35)	0.26 (0.21–0.37)	0.30 (0.26–0.34)	1.000	1.000	1.000	1.000	1.000	1.000
	Day 7	0.33 (0.26–0.38)	0.28 (0.24–0.39)	0.18 (0.17–0.32)	0.31 (0.27–0.35)	1.000	0.716	1.000	1.000	1000	1.000
	Day 14	0.32 (0.24–0.40)	0.29 (0.23–0.34)	0.22 (0.18–0.32)	0.27 (0.23–0.34)	1.000	0.877	1.000	1.000	1.000	1.000
Kep (10^−3^ min^-1^)	Day 0	1.38 (1.17–2.02)	1.49 (1.10–2.10)	1.45 (1.25–2.43)	1.73 (1.41–2.18)	1.000	1.000	1.000	1.000	1.000	1.000
	Day 7	0.66 (0.46–1.08)	1.22 (0.81–1.85)	1.60 (1.03–3.06)	1.59 (1.29–2.41)	0.933	0.005	0.039	0.287	1.000	1.000
	Day 14	0.64 (0.26–0.89)	1.14 (0.69–1.58)	1.47 (1.07–2.54)	1.63 (1.25–2.28)	1.000	0.017	0.048	0.437	0.846	1.000
Vp	Day 0	0.06 (0.03–0.15)	0.07 (0.03–0.21)	0.10 (0.06–0.12)	0.12 (0.04–0.14)	1.000	1.000	1.000	1.000	1.000	1.000
	Day 7	0.09 (0.03–0.23)	0.08 (0.04–0.17)	0.15 (0.09–0.17)	0.08 (0.05–0.14)	1.000	1.000	1.000	1.000	1.000	1.000
	Day 14	0.10 (0.03–0.17)	0.11 (0.03–0.17)	0.10 (0.06–0.21)	0.14 (0.12–0.21)	1.000	1.000	1.000	1.000	1.000	1.000

Data are presented as median (IQR).

^a^ p-values are obtained through repeated-measures analysis of variance with post-hoc analyses using Bonferroni correction.

Changes in ADC and DCE parameters were observed in the IA-T group compared to those in the IA-S and Sham control groups. ADC was significantly higher in the IA-T group on day 14 (7.62 [IQR 7.23–8.20] vs. IA-S, 6.77 [IQR 6.48–6.87] vs. Sham control 6.89 [4.93–7.48], p = 0.044 and p = 0.004, respectively). The Ktrans and Kep of the IA-T group significantly decreased on day 14 when compared to those of the IA-S and Sham control groups. Ktrans and Kep decreased by 59.1% and 56.9%, respectively, in the IA-T group on day 14. In the IV-T group, Ktrans and Kep decreased by 19.1% and 19.2%, respectively, on day 7; however, this difference was insignificant. The DCE parameters did not change significantly in the other three groups on day 14.

**Fig 4:**
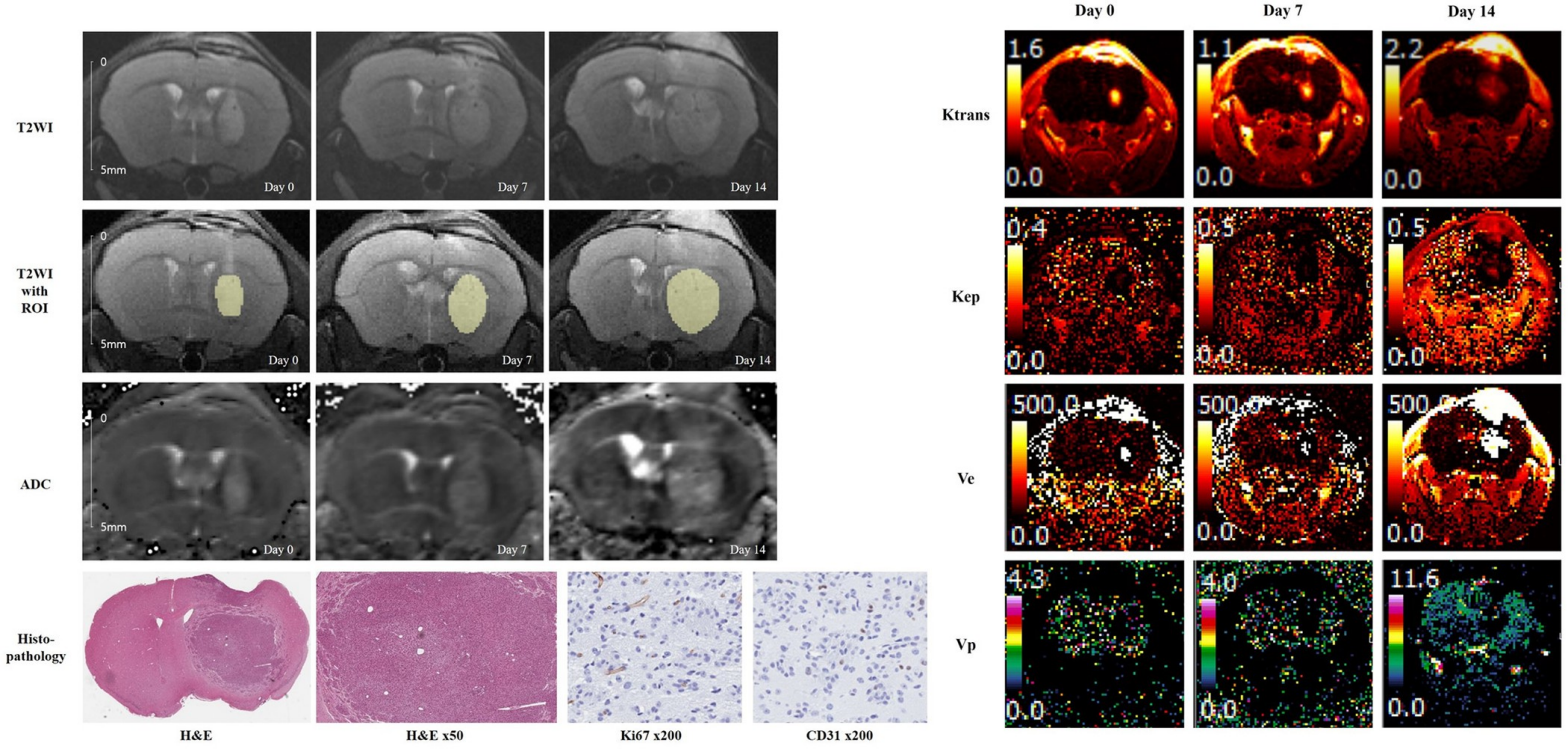
Representative cases of the BT474 xenograft model treated with intra-arterial trastuzumab injections. Pretreatment T2 weighted image (day 0) showing a mildly hyperintense tumor with serial growth on follow-up MRI. On day 14, the ADC value of the tumor increased by 11.3%, and Ktrans decreased by 59.1% compared to that at baseline. Hematoxylin and eosin-stained slides showed reduced cellularity at the periphery of the tumor. The Ki-67 index was 7.9%.

**Fig 5:**
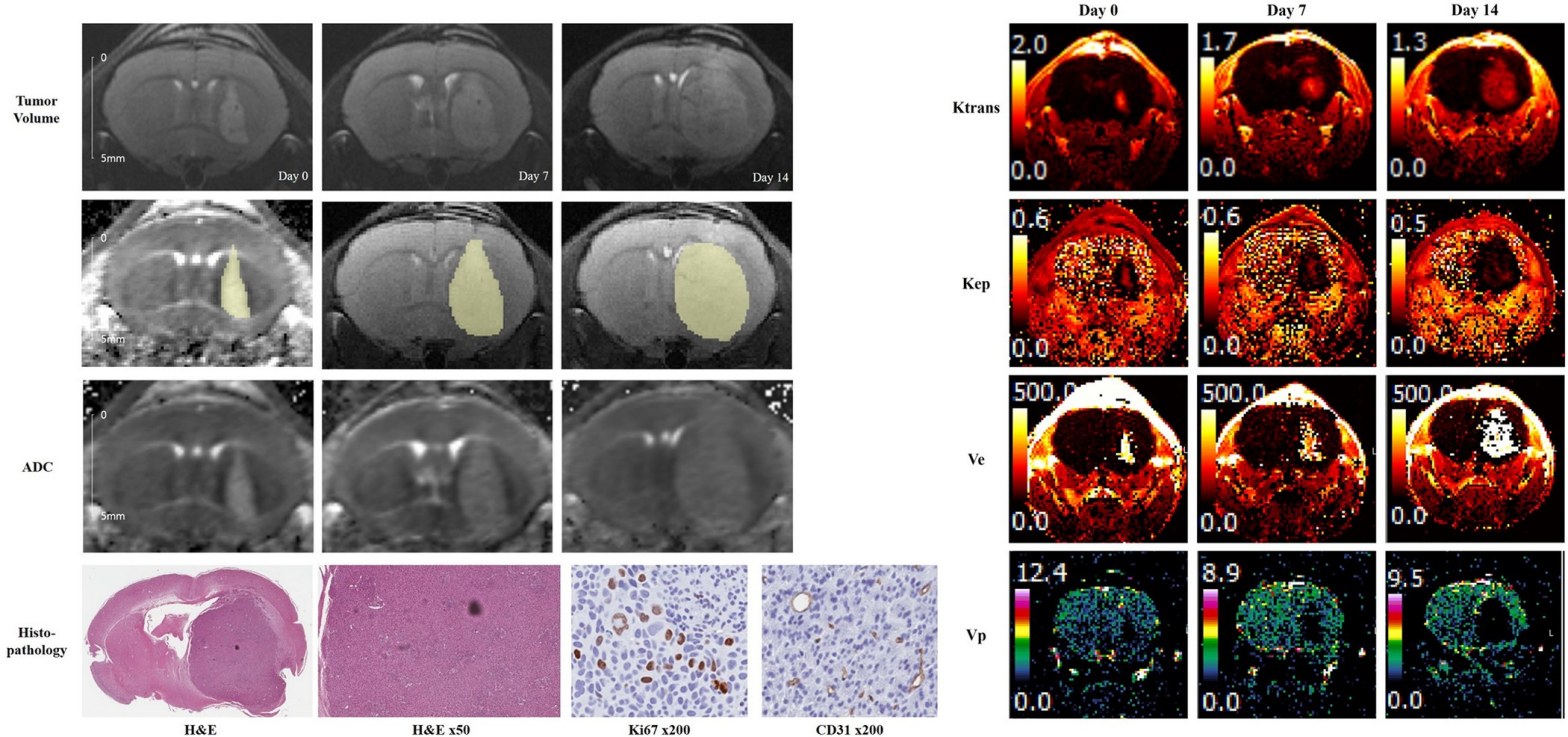
Representative cases of the BT474 xenograft model treated with intra-arterial saline injection. Pretreatment T2 weighted image showed aggressive tumor growth with a significant mass effect. The ADC value and Ktrans on day 14 decreased by only 4.5% and 2.2%, respectively. Hematoxylin-eosin-stained slides showed compact cellularity with an increased vascular structure. The Ki-67 index was 14.8%.

Histopathological examination demonstrated that the IA-T group exhibited less cellularity and lower Ki67 and CD34 expression than the IA-S group. (Figs 4 and 5).

## Discussion

IA treatment caused tumor growth reduction and a significant change in initial imaging parameters compared to those in the IV treatment and non-treatment groups. An increase in the ADC value suggested a decrease in cellularity due to tumor growth inhibition, and a decrease in Ktrans and Kep could be explained by a decrease in the diffusion and perfusion coefficients due to the inhibition of angiogenesis. In addition, the initial response of DCE-MRI parameters is related to tumor response to future treatment; these findings suggest that subsequent treatment could proceed successfully [18, 22].

Anti-tumor effects are based on drug dose and tumor sensitivity and are related to the route of drug delivery. IA administration positively affects recurrent or progressive malignant glioblastomas, retinoblastomas, and primary CNS lymphomas [23–25]. Trastuzumab for metastatic breast cancer is used in animal xenograft models and clinical practice; however, there is a lack of data on IA injection [19, 25]. Therefore, we compared the treatment response to intra-arterial injection with that to IV treatment and compared it with that observed in the IA-S group to confirm the adverse effects of intra-arterial injection itself. Significant reductions in tumor growth and changes in ADC and DCE parameters were observed in the IA-T group compared to those in the non-treatment group; these changes were not observed in the IV-T group.

Contrast enhancement in brain tumors indicates an increase in vascular permeability due to the breakdown of the BBB. However, the presence of enhancement limits evaluation, especially differentiating between tumor recurrence and treatment changes. Therefore, multimodal MRI, including DWI and DCE, is widely used to accurately assess and predict the tumor status following anti-cancer treatment. DCE provides information on the hemodynamic changes in tumors and measures the properties of the microvascular structure and permeability in tumors. Although Ktrans can be affected by blood flow when the condition with disrupted BBB and may not solely reflect vascular permeability, Ktrans acts as a functional parameter for measuring the transport rate of the contrast agent from the blood plasma to the extravascular–extracellular space. In glioblastomas, contrast leakage increases because of the disruption of the BBB of pre-existing blood vessels or the imperfect BBB of abnormal vessels from angiogenesis. Increased Ktrans values are associated with higher-grade glioma, tumor recurrence, and a worse prognosis in patients with glioblastoma [26, 27]. In addition, brain metastasis showed a DCE parameter response similar to that of glioblastoma; higher Ktrans in early post-radiosurgery imaging can predict a poor long-term response [28]. Trastuzumab has an anti-angiogenic effect; decreased vascular permeability and density following treatment can be attributed to decreased Ktrans.

The ADC value is a useful marker, well documented in various studies. Low ADC values reflect malignancy in tissues with high cellularity [29]. However, acute ischemic stroke can contribute to changes in ADC value; ADC decreases due to cytotoxic edema during the acute phase of ischemic stroke and increases due to vasogenic edema beyond acute phase. Therefore, we investigated two control groups to evaluate the effect of carotid artery ligation on ADC changes. In this study, there was no significant ADC difference between the IA-S and sham control groups; therefore, the ADC change due to cerebral ischemia was insignificant.

Trastuzumab is generally known to have poor penetration into the brain. However, the BBB potentially allows larger molecules penetrate the brain parenchyma, because it disrupted at the site of metastases. Previous study shows that 89Zr-trastuzumab can target brain lesions, with an 18-fold higher uptake in tumors than in normal brain tissue [30]. Further, increased local concentration of trastuzumab also related to inducing cell apoptosis, resulting further increase of BBB disruption.

This study had several limitations due to the limited number of animals and conditions. In the in vivo experiments, additional IA injection was impossible because the carotid artery was ligated after the intra-arterial injection, and fibrosis was observed during the healing of the dissection site. MRI parameters reflect the early response of the tumor; however, further evaluation is mandatory to evaluate the actual treatment effect. In the IA treatment group, the number of subjects that died before day 14 was slightly higher than that in the other groups. The MRI showed no evidence of cerebral infarction or edema; however, the adverse effects of carotid ligation after IA injection could not be excluded. Further, ischemic changes may also be related to the BBB breakdown, which increase permeability and influence DCE parameter changes. Also, we excluded five mice that showed excessive tumor growth it may introduce biases. However, excessive tumor growth was not seen in the IA-T group, so the impact on the conclusion will be limited. We used population-based AIF; therefore, there were limitations in reflecting differences according to the variations between subjects. To measure the AIF in each subject, sufficient temporal and spatial resolution must be secured in the distal internal carotid artery; this requires further investigation. Finally, because of small size of tumor and limited signal-noise ratio, it was limited to completely eliminate internal necrosis from ROI. Further, although tumors in our study showed well-defined margin on T2WI, manual ROI based analysis may produce numerical error.

In conclusion, IA treatment using trastuzumab has a potential effect on the early response to treatment, including tumor growth reduction, decreased cellularity, and decreased Ktrans in preclinical brain tumor models. Further studies are needed to evaluate the actual tumor response and clinical efficacy. IA treatment showed positive changes in MRI parameters, promising a good response to further treatment.

## Supporting information

S1 Fig Cell viability for BT474, ZR75-1, and SKBR3 treated with trastuzumab. BT474 was considered the most sensitive cell line, as more than 30% of cell death observed for 1 μg/mL trastuzumab.
(TIF)

S2 Fig Preliminary study for confirming tumor growth of each cell line. T2 weighted images obtained 4 weeks after stereotactic tumor cell injection demonstrated hyperintense tumor mass in BT474 xenograft model. Tumor size was too small to measure in the SKBR3 and ZR75-1 xenograft model.
(JPG)

S1 File Humane endpoint of this study. (DOCX)

S2 File MRI images and tumor volume measurement of representative cases. (PPTX)

S3 File Population based AIF measurement. (DOCX)
