# Supplementary figures and images for: Early therapy evaluation of intra-arterial trastuzumab injection in a human breast cancer xenograft model using multiparametric MR imaging
Source: PLoS One. 2024 May 3;19(5):e0300171. doi: 10.1371/journal.pone.0300171 (PMC11068173; doi:10.1371/journal.pone.0300171)

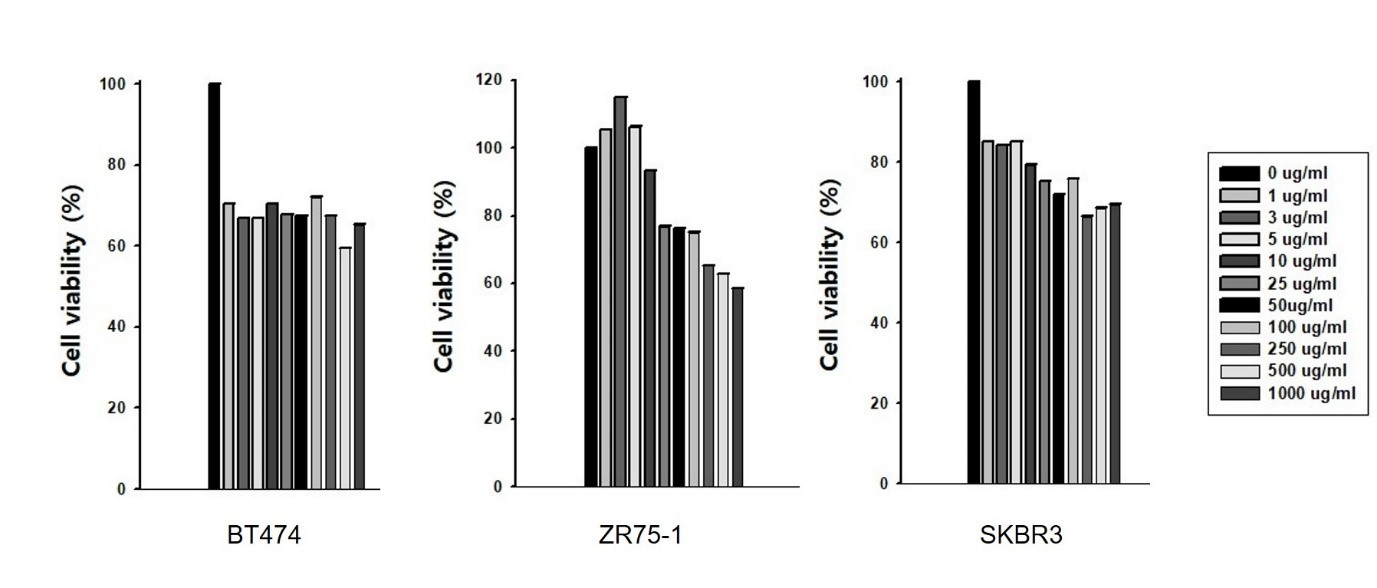

Supplement: S1 Fig — BT474 was considered the most sensitive cell line, as more than 30% of cell death observed for 1 μg/mL trastuzumab. (TIF) [file pone.0300171.s001.tif]

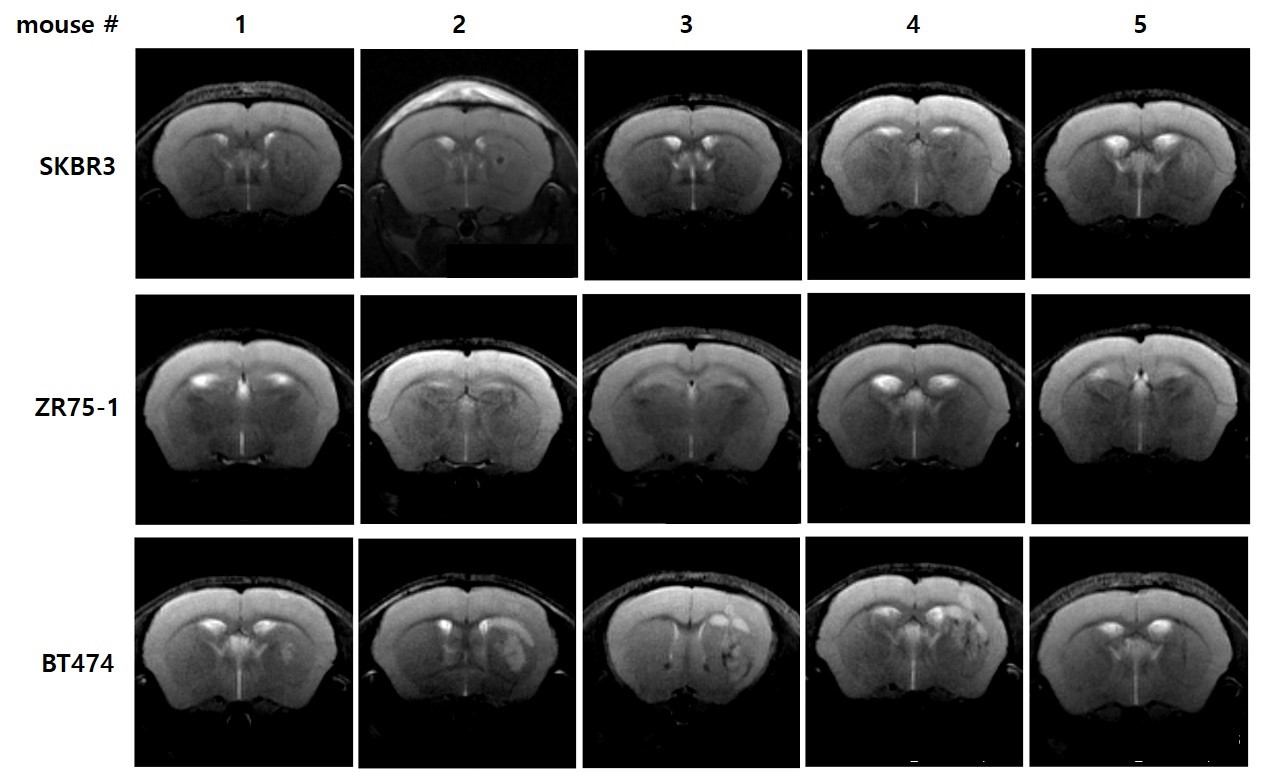

Supplement: S2 Fig — T2 weighted images obtained 4 weeks after stereotactic tumor cell injection demonstrated hyperintense tumor mass in BT474 xenograft model. Tumor size was too small to measure in the SKBR3 and ZR75-1 xenograft model. (JPG) [file pone.0300171.s002.jpg]
