# Supplementary material for: Early therapy evaluation of intra-arterial trastuzumab injection in a human breast cancer xenograft model using multiparametric MR imaging
Source: PLoS One. 2024 May 3;19(5):e0300171. doi: 10.1371/journal.pone.0300171 (PMC11068173; doi:10.1371/journal.pone.0300171)
Supplement: S1 File — (DOCX) [file pone.0300171.s003.docx]

Humane endpoint

Humane endpoints were composed of body weight growth (body weight loss > 30%), responsiveness (delayed and null response or no response), appearance (dilated pupil or abnormal posture), respiration (labored breathing or gasping), and other clinical signs (seizures, failure to eat or drink). We assessed and monitored the condition of animals in a daily basis. If an individual showed more than three or more categories, it was determined as humane endpoint. Because the tumor was implanted in the brain, the largest dimension of the tumor did not exceed 5 mm, so tumor size was considered to be unsuitable as an endpoint, and was instead evaluated by clinical signs described above due to mass effect. Euthanasia was planned to be performed via a deep pentobarbital (Entobar®, Hanlim Pharmaceutical C7-8o., Seoul, Korea) anesthesia (60 mg/kg, intraperitoneal).
