# Supplementary material for: Early therapy evaluation of intra-arterial trastuzumab injection in a human breast cancer xenograft model using multiparametric MR imaging
Source: PLoS One. 2024 May 3;19(5):e0300171. doi: 10.1371/journal.pone.0300171 (PMC11068173; doi:10.1371/journal.pone.0300171)
Supplement: S2 File — (PPTX) [file pone.0300171.s004.pptx]

## Slide 1
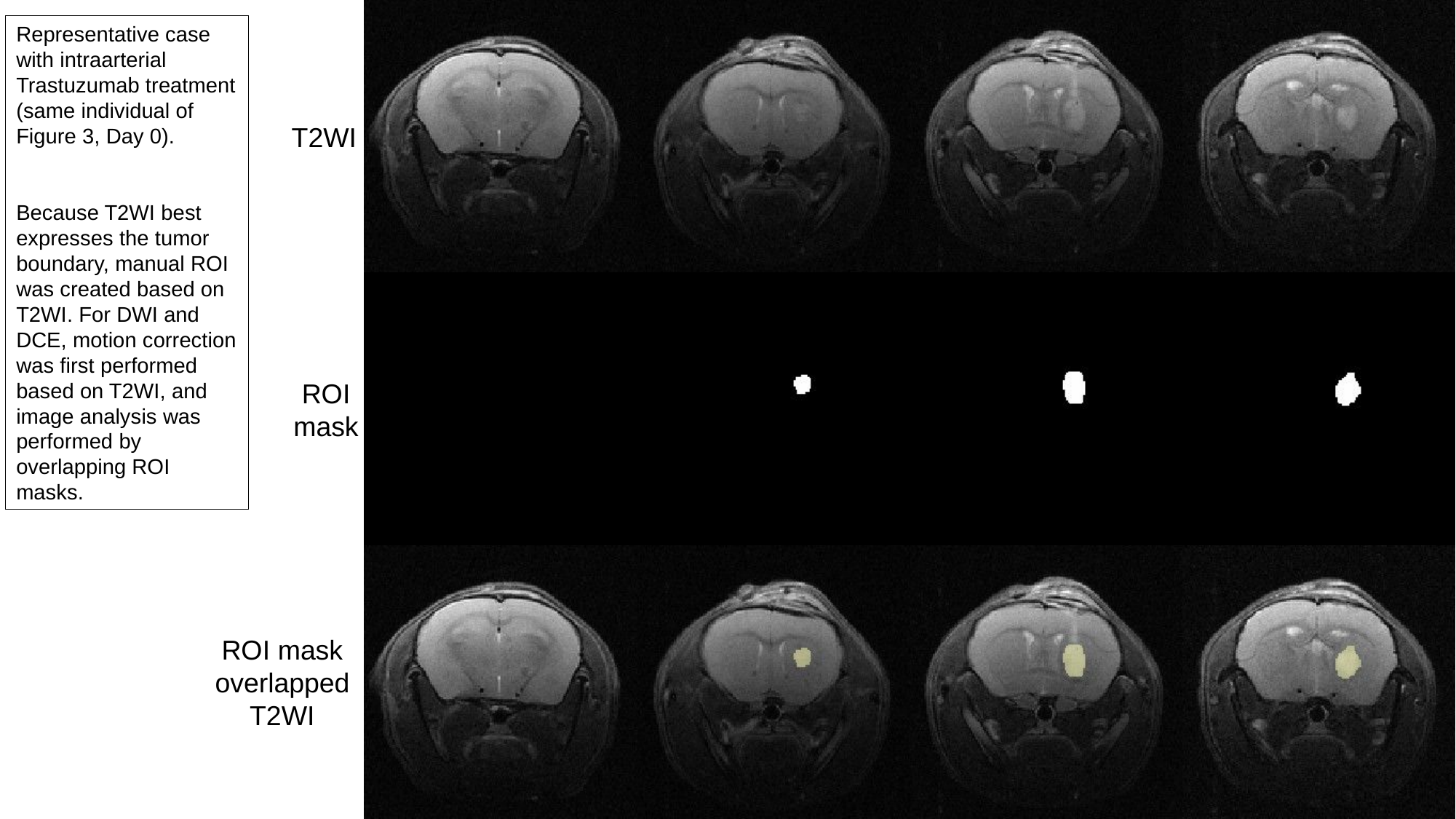

Representative case with intraarterial Trastuzumab treatment (same individual of Figure 3, Day 0).
Because T2WI best expresses the tumor boundary, manual ROI was created based on T2WI. For DWI and DCE, motion correction was first performed based on T2WI, and image analysis was performed by overlapping ROI masks.
T2WI
ROI mask
ROI mask overlapped T2WI

## Slide 2
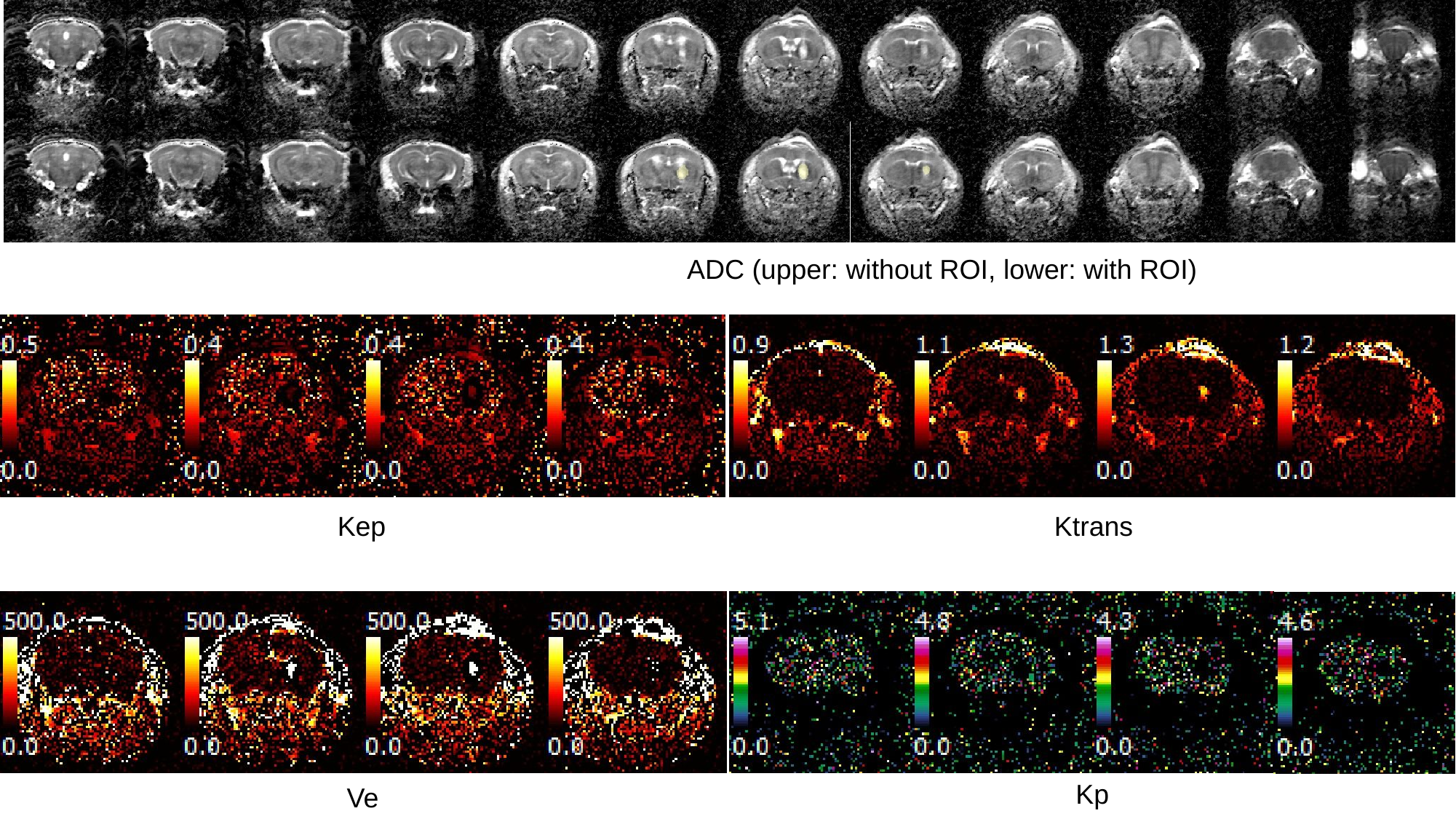

ADC (upper: without ROI, lower: with ROI)
Kep
Ktrans
Kp
Ve

## Slide 3
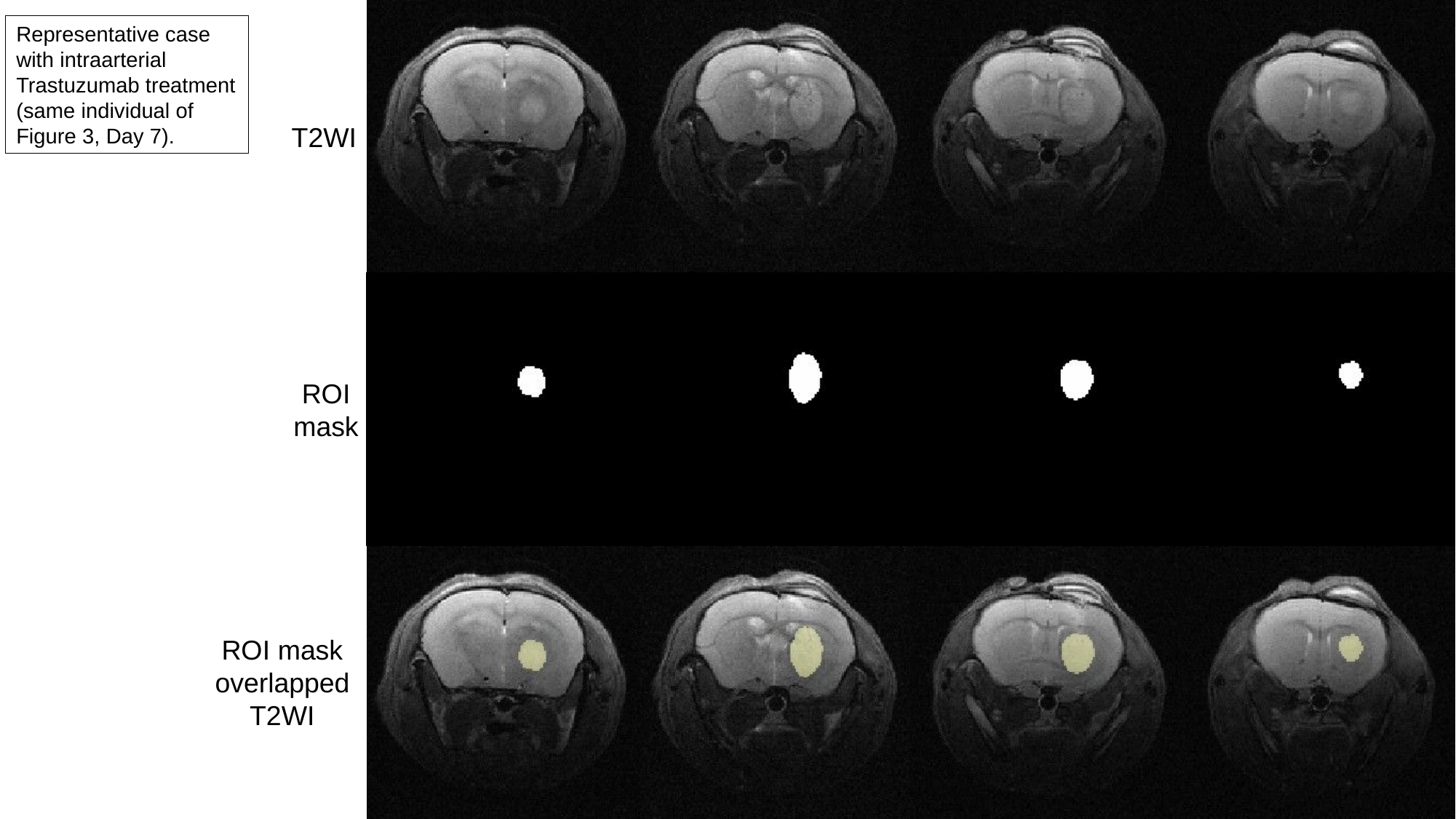

Representative case with intraarterial Trastuzumab treatment (same individual of Figure 3, Day 7).
T2WI
ROI mask
ROI mask overlapped T2WI

## Slide 4
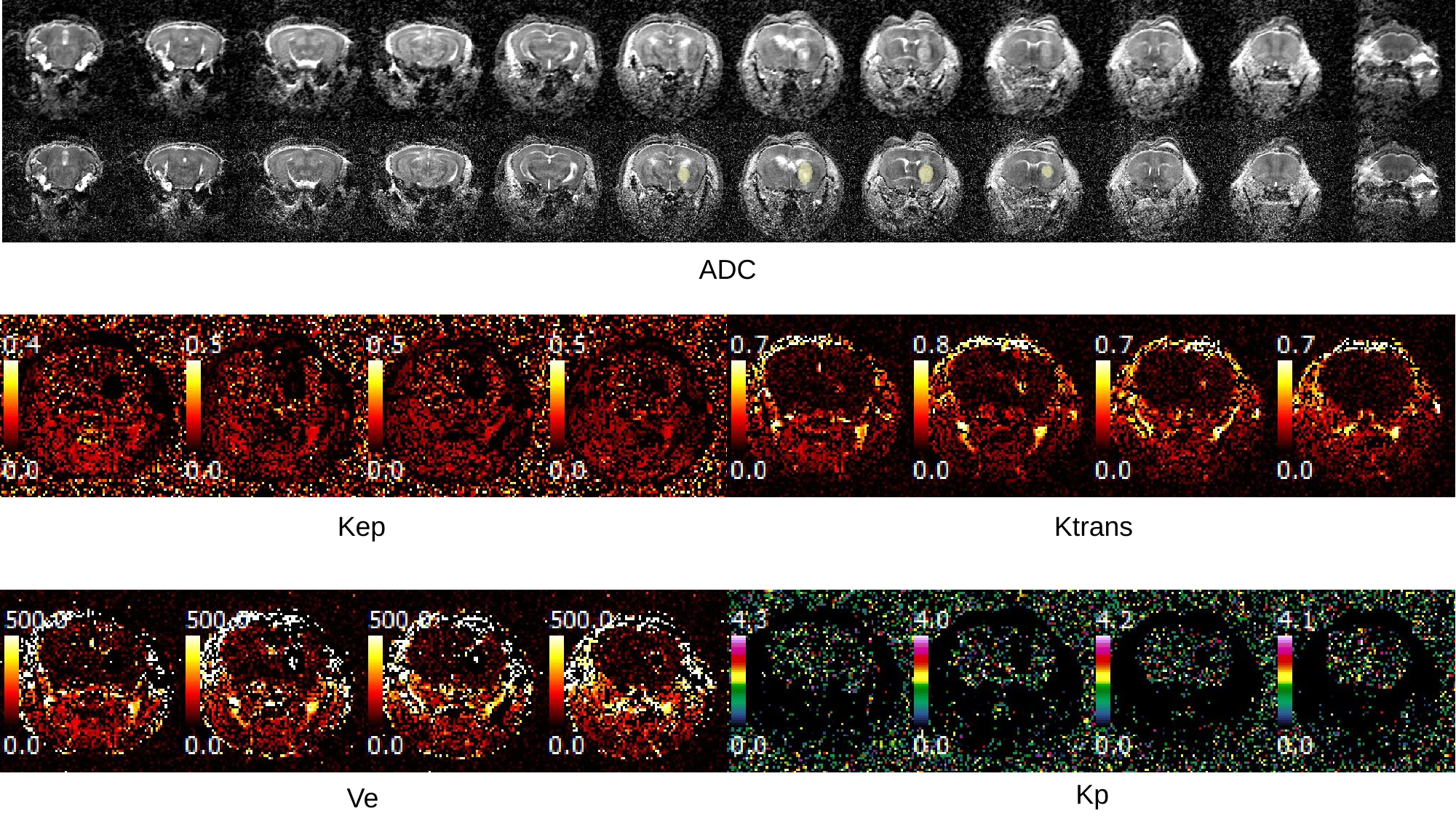

ADC
Kep
Ktrans
Kp
Ve

## Slide 5
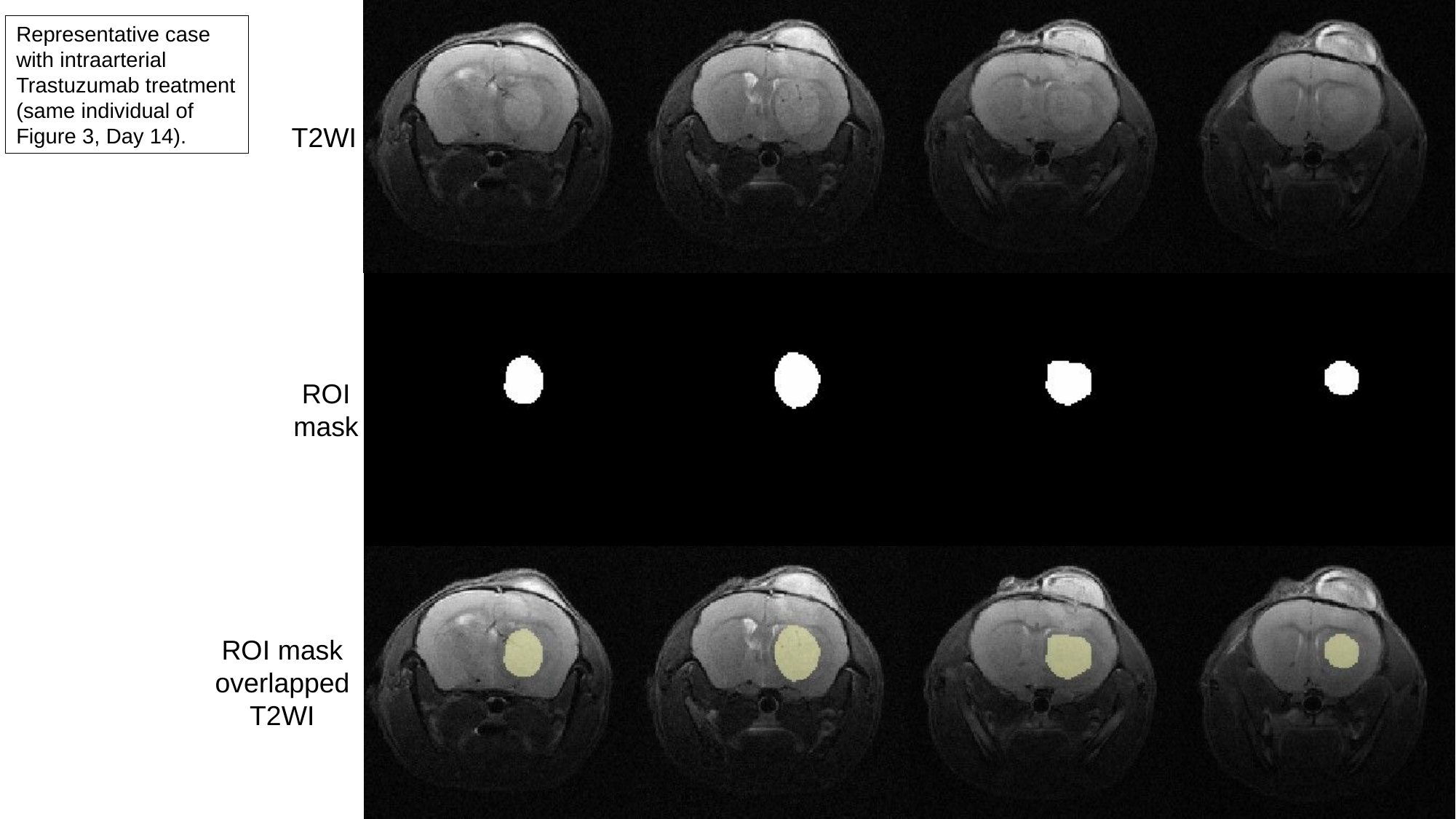

Representative case with intraarterial Trastuzumab treatment (same individual of Figure 3, Day 14).
T2WI
ROI mask
ROI mask overlapped T2WI

## Slide 6
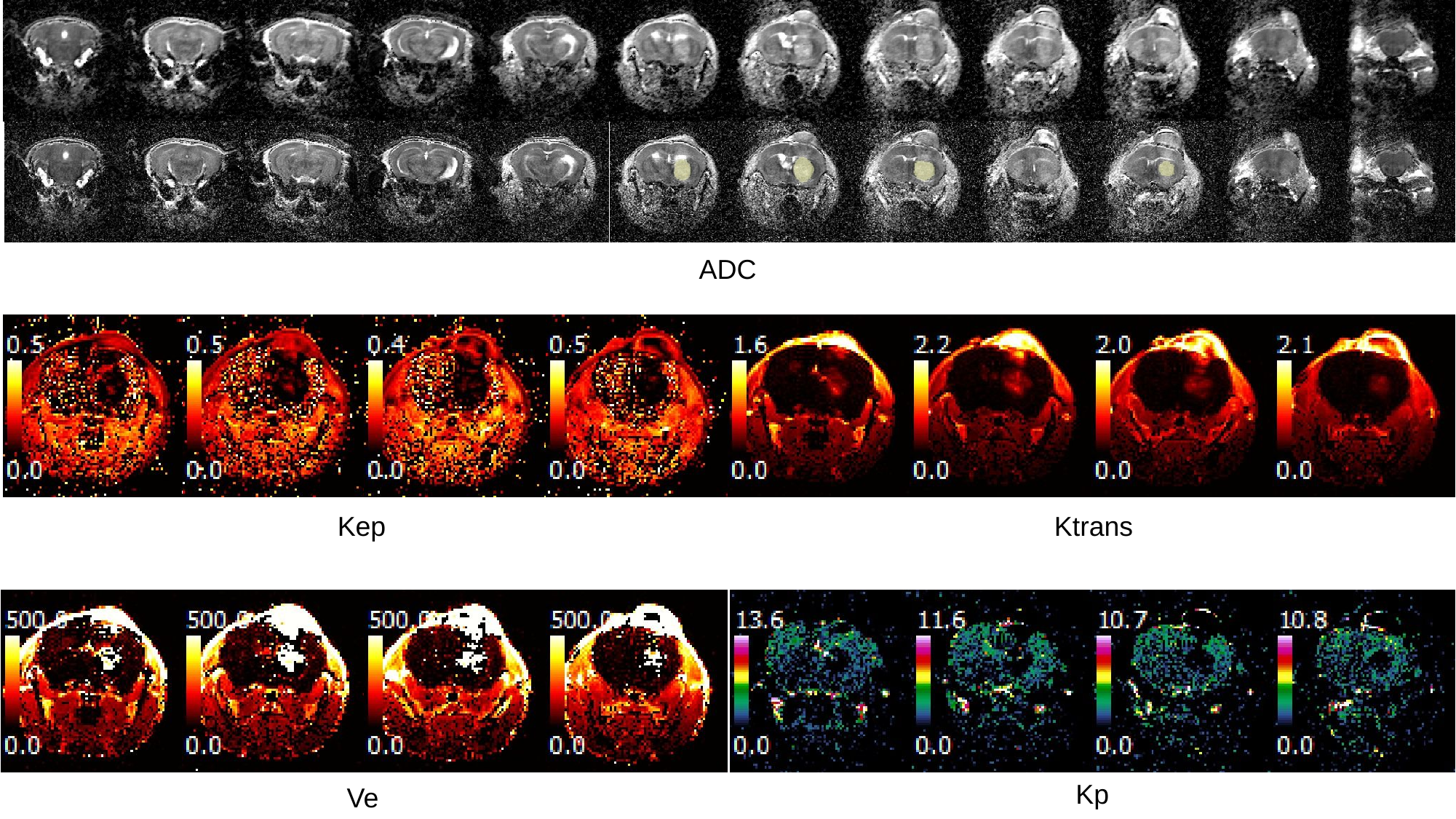

ADC
Kep
Ktrans
Kp
Ve

## Slide 7
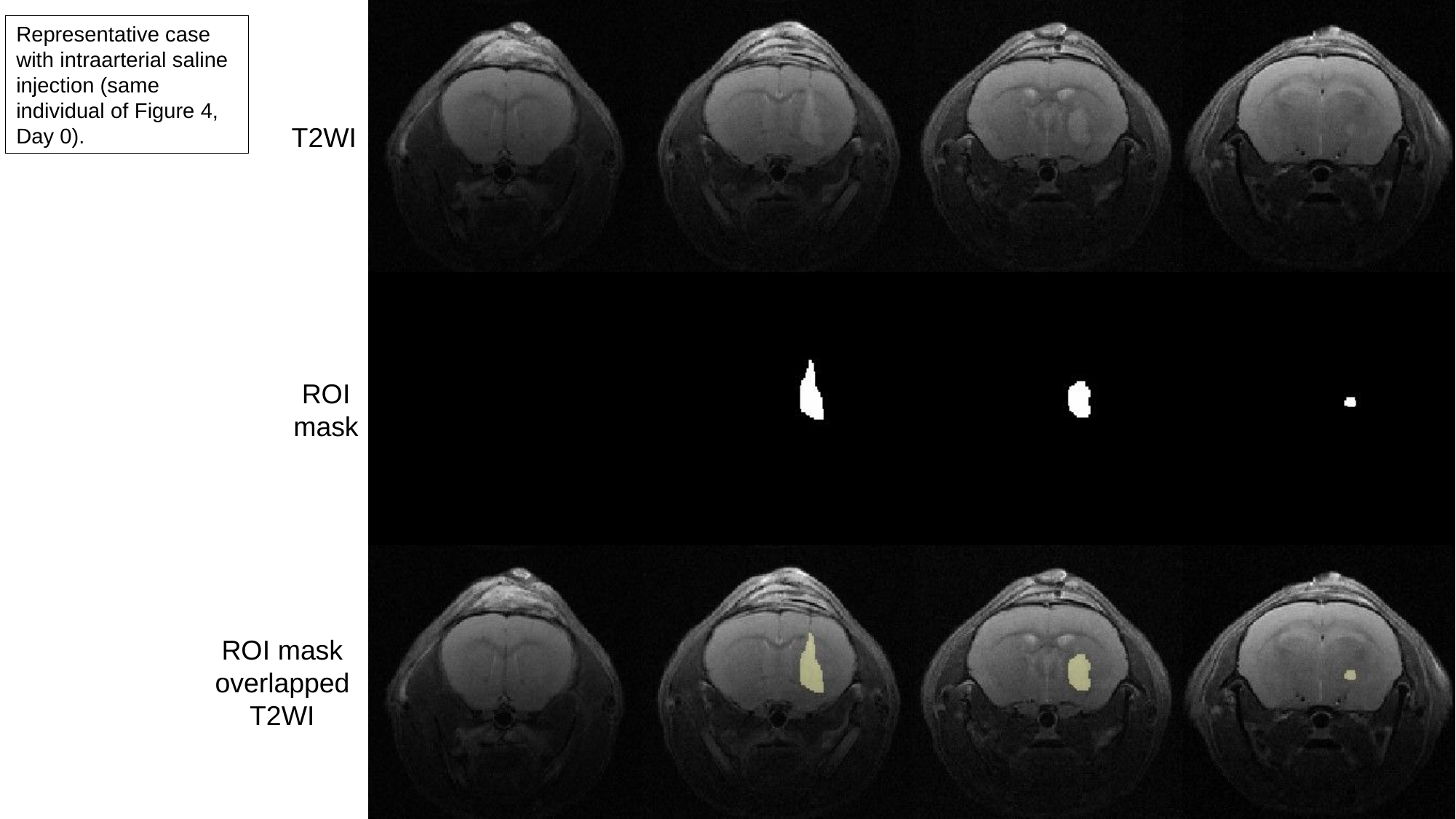

Representative case with intraarterial saline injection (same individual of Figure 4, Day 0).
T2WI
ROI mask
ROI mask overlapped T2WI

## Slide 8
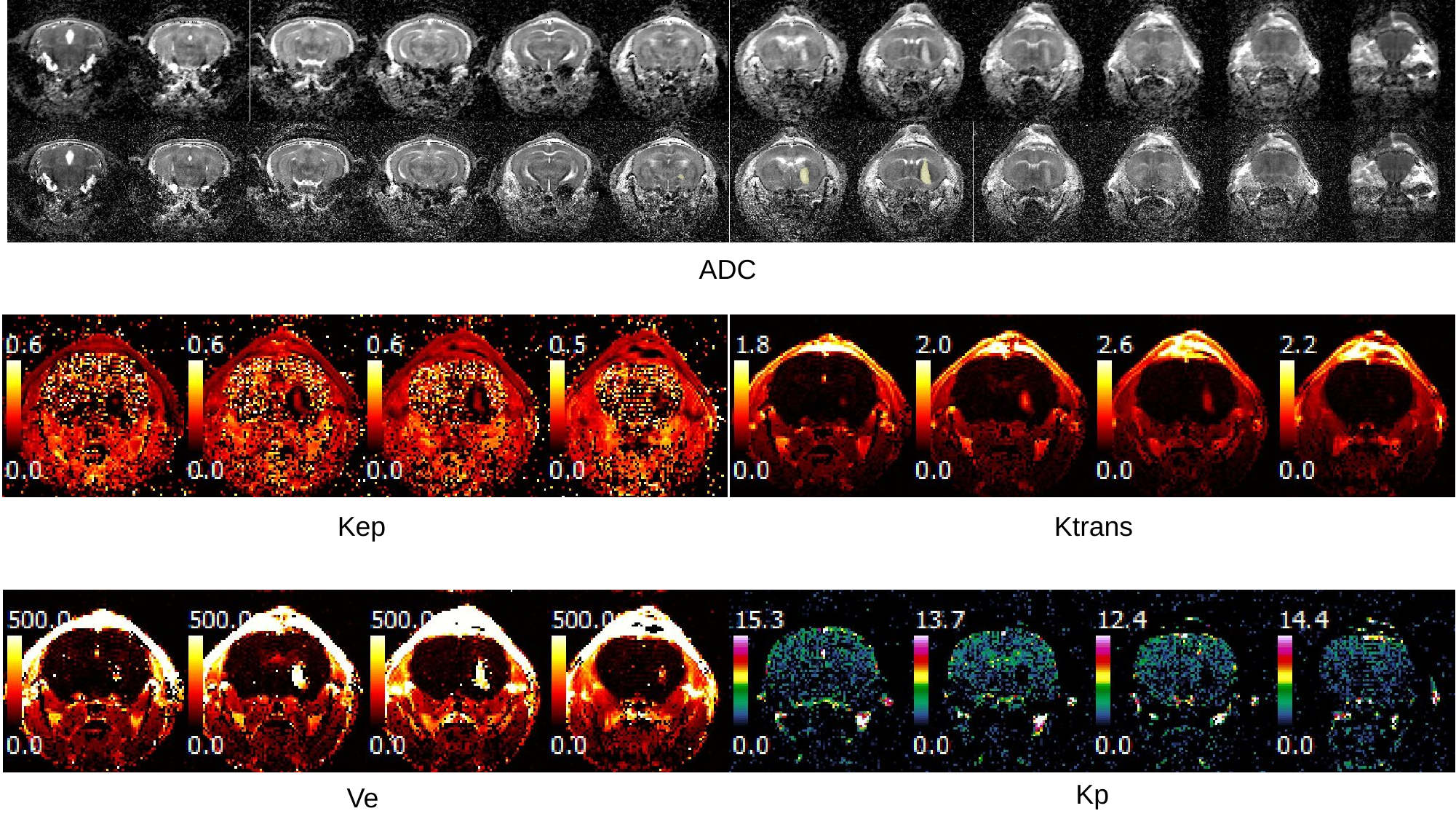

ADC
Kep
Ktrans
Kp
Ve

## Slide 9
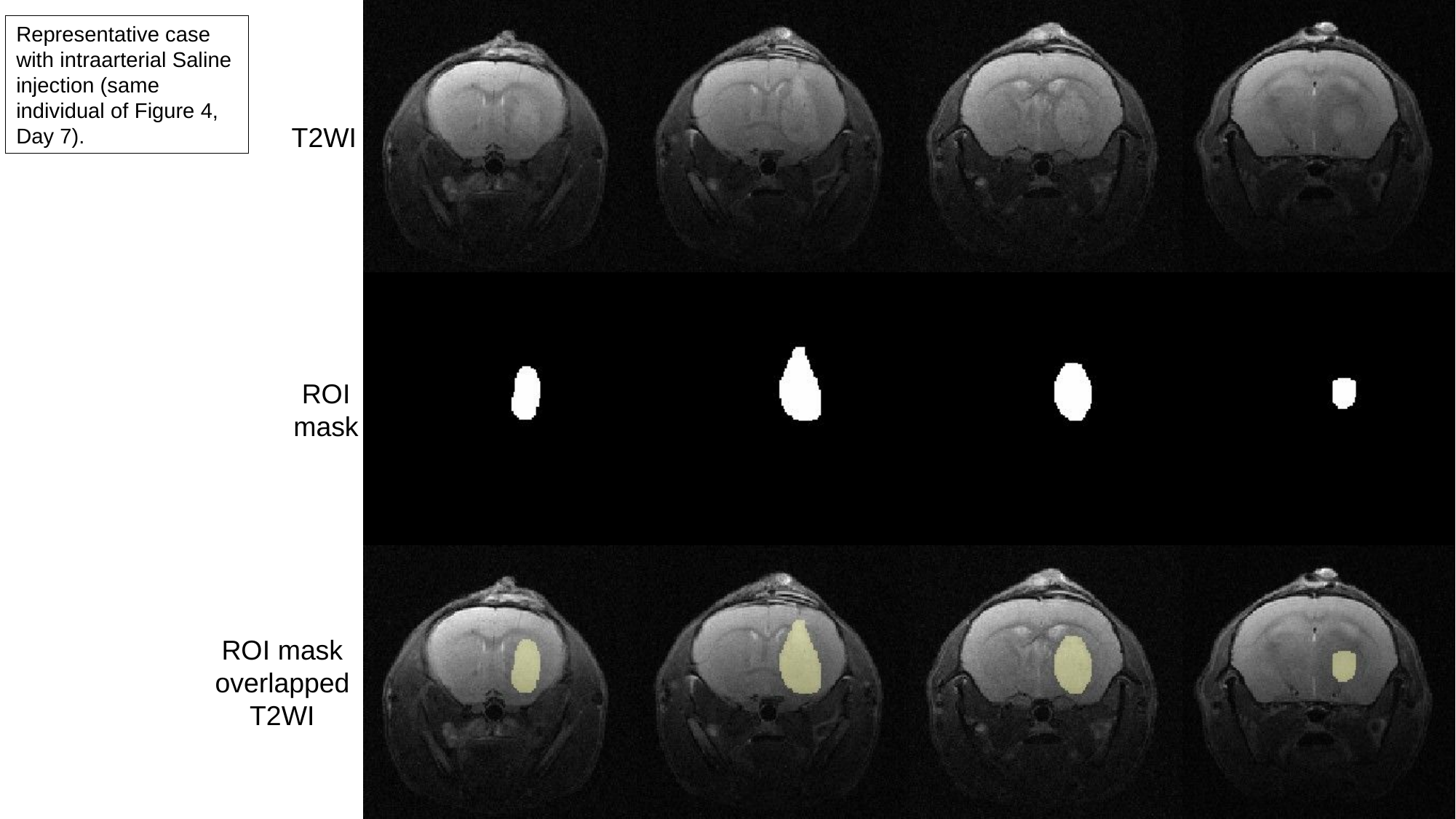

Representative case with intraarterial Saline injection (same individual of Figure 4, Day 7).
T2WI
ROI mask
ROI mask overlapped T2WI

## Slide 10
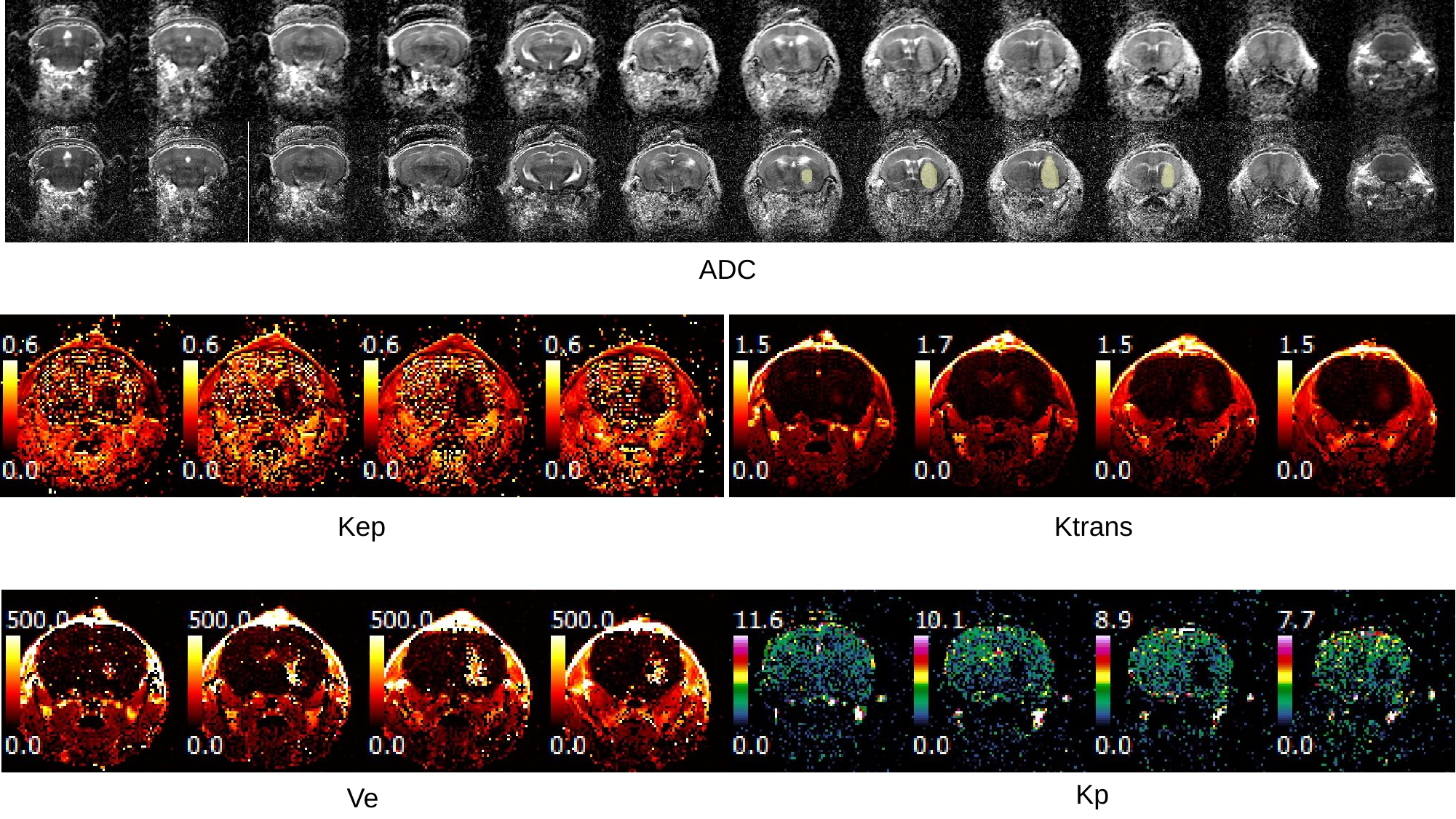

ADC
Kep
Ktrans
Kp
Ve

## Slide 11
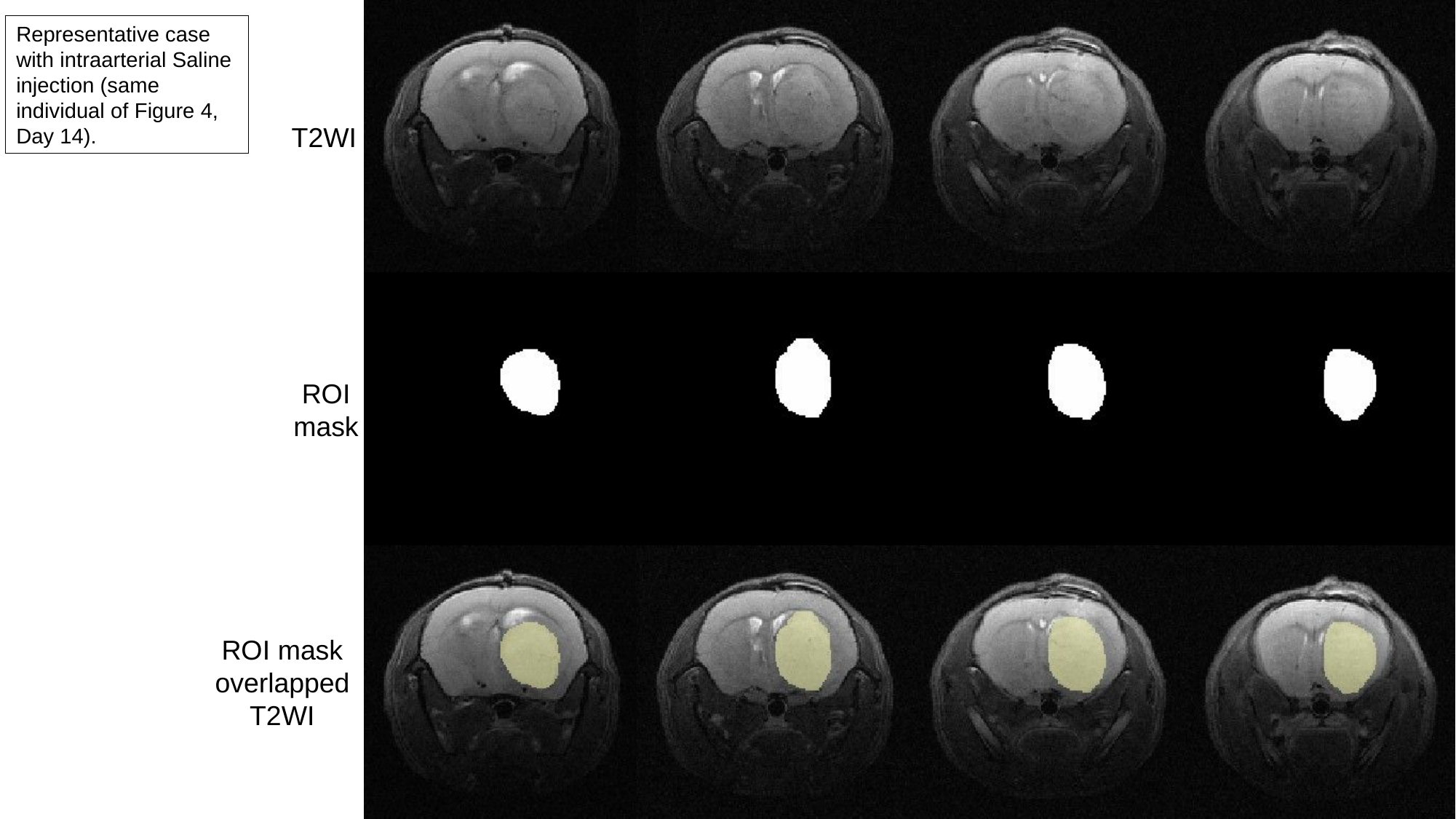

Representative case with intraarterial Saline injection (same individual of Figure 4, Day 14).
T2WI
ROI mask
ROI mask overlapped T2WI

## Slide 12
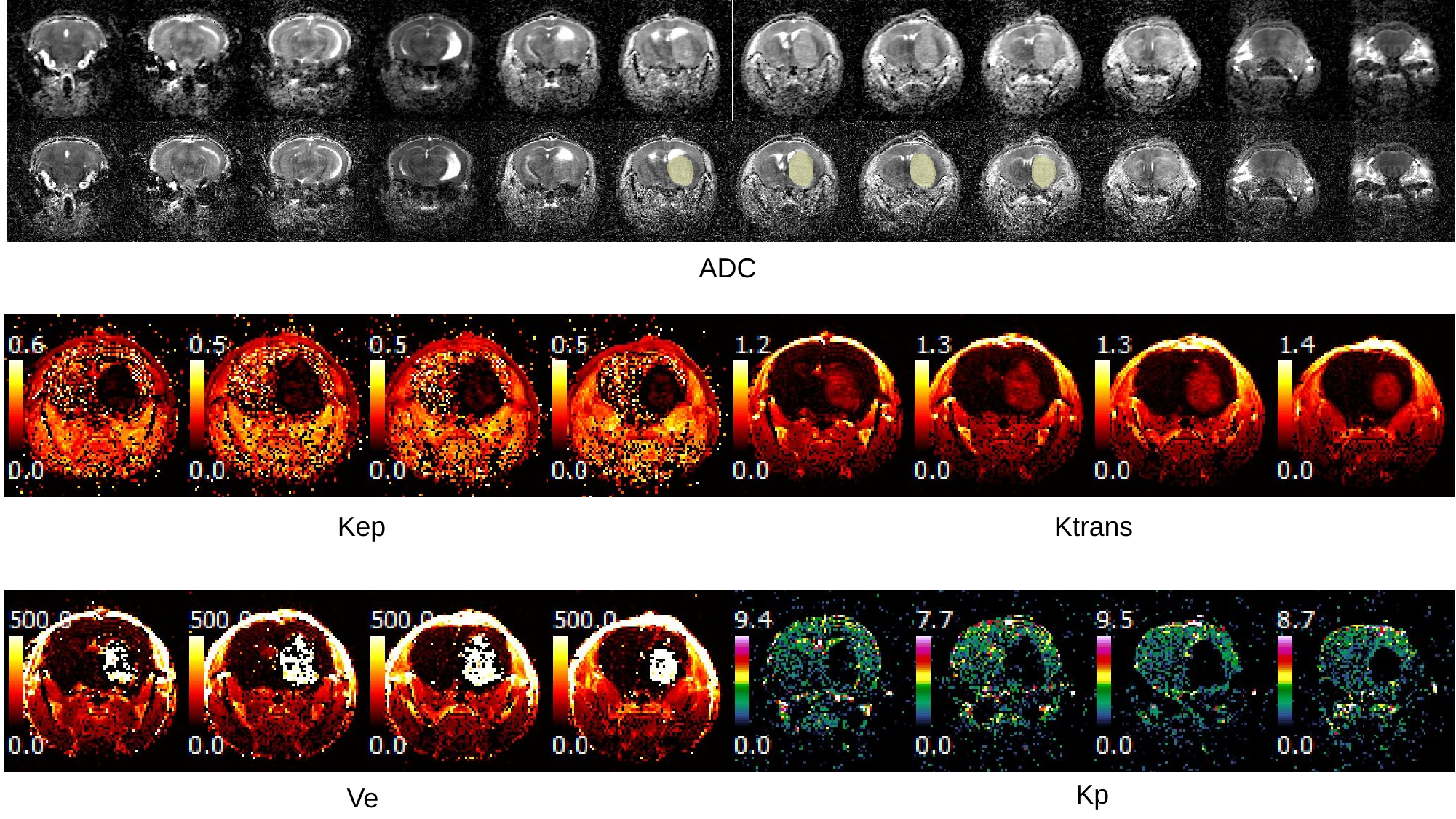

ADC
Kep
Ktrans
Kp
Ve
