# Supplementary material for: Early therapy evaluation of intra-arterial trastuzumab injection in a human breast cancer xenograft model using multiparametric MR imaging
Source: PLoS One. 2024 May 3;19(5):e0300171. doi: 10.1371/journal.pone.0300171 (PMC11068173; doi:10.1371/journal.pone.0300171)
Supplement: S3 File — (DOCX) [file pone.0300171.s005.docx]

Population based AIF measurement

To establish population-based AIF, we performed DCE-MRI at a separate cohort of five tumor bearing mice with the same parameters. The location of the distal internal carotid artery was determined by referring to the image of the early arterial phase during DCE and the darkSI seen by the flow void in the T2 weighted image. A region of interest (ROI), consists of 5 pixels, was carefully drawn within the boundaries of the ICA. The temporal AIF concentrations were acquired and the mean spatial amplitude was averaged. (Supplementary Figure 1) The averaged concentration curve was fitted to the bi-exponential model (1) yielding the amplitude and decay rates for general kinetic model.

Cp(t) = A1e^−m1t^ + A2e^−m2t^ (1)

(Cp: the tracer concentration in the blood plasma, t: time, Ai and mi (i =1, 2): the amplitude and decay rates)


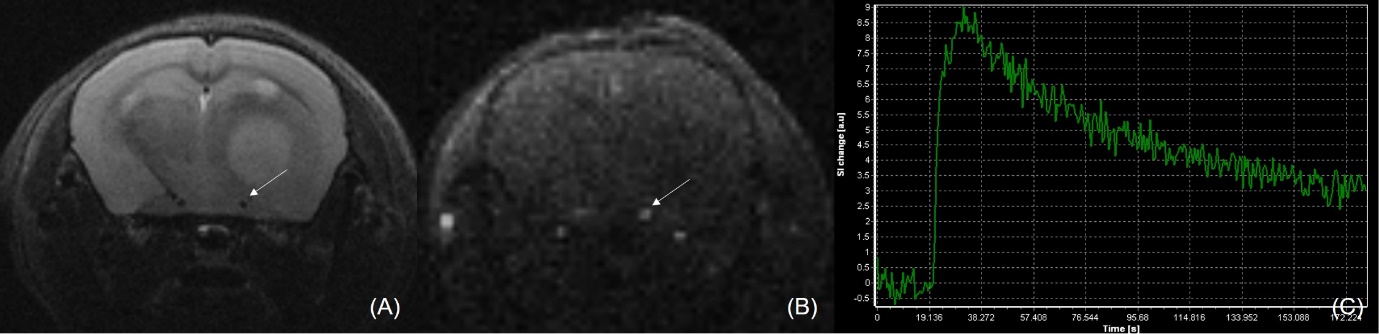


Supplementary Figure 1. T2-weighted image (A) and contrast-enhanced T1-weighted image (B) shows distal ICA (arrow). After region of ROI that consists of 5 pixels was placed at the location of distal ICA, the average concentration curve (C) was obtained using imaging analysis software (Nordic ICE, NordicNeuroLab, Bergen, Norway).
